# 

**Published:** 1915-06

**Authors:** 


					﻿The Package Library gives access to the best in $500.00 worth of medical journals
Vol. XXX
JUNE, 1915
No. 12
TE
Medical Journal
“Red Back”
ESTABLISHED JOLT, 1885
PUBLISHEP MONTHLY AT^ AUSTIN, TEXAS, BY
MRS. F. E. DANIEL,	Publisher and Managing Editor
.Subscription, 81.00 a Year, in Advance.’ «	- Single Copies,10Cents
Entered at the Ppstoffice at Austin, Texas, as second class mail matter.
				

